# Supplementary material for: Scalable Wettability Modification of Aluminum Surface through Single-Shot Nanosecond Laser Processing
Source: Nanomaterials (Basel). 2023 Apr 17;13(8):1392. doi: 10.3390/nano13081392 (PMC10145128; doi:10.3390/nano13081392)
Supplement: Supplementary file 1 [file nanomaterials-13-01392-s001.zip › Supporting information.pdf]

SUPPLEMENTARY INFORMATION

Scalable Wettability Modification of Aluminum Surface through Single-Shot Nanosecond Laser Processing

Chi-Vinh Ngo 1,†, Yu Liu 1,2,†, Wei Li 1,2, Jianjun Yang 1,* and Chunlei Guo 3,*

[1] GPL Photonics Laboratory, State Key Laboratory of Luminescence and Applications, Changchun Institute of Optics, Fine Mechanics and Physics, Chinese Academy of Sciences, Changchun, Jilin 130033, China
[2] University of Chinese Academy of Science, Beijing 100049, China
[3] The Institute of Optics, University of Rochester, Rochester, New York 14627, USA
* Correspondence: jjyang@ciomp.ac.cn (J.Y.); guo@optics.rochester.edu (C.G.)
† These authors contributed equally to this work.

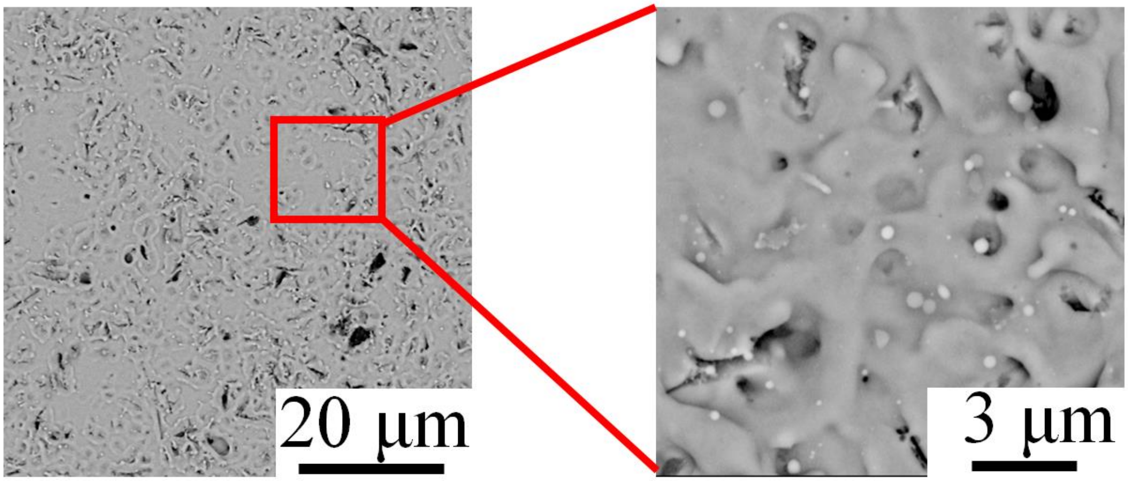

Figure S1. SEM images of the stainless-steel surface after the single-shot irradiation with the energy of 390 mJ.

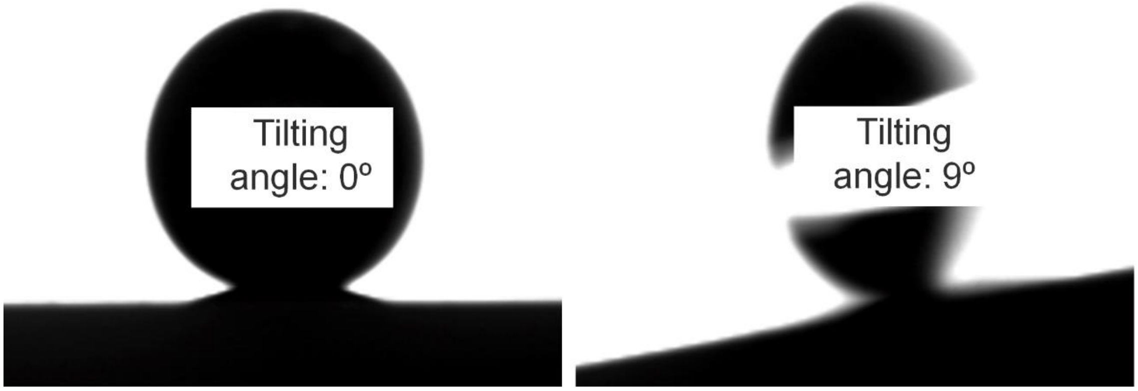

Figure S2. The measured sliding angle of the aluminum target after the single-shot nanosecond laser irradiation at the pulse energy of 192 mJ. The water droplet started to slide off the aluminum surface at a tilting angle of 9°.

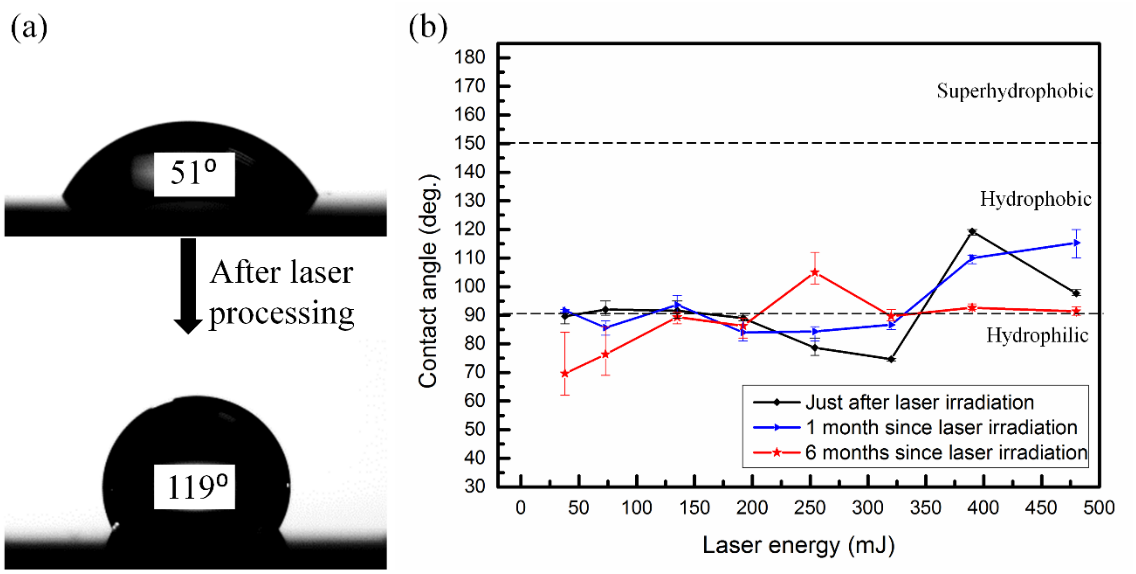

Figure S3. (a) Wettability of stainless-steel surface after the single-shot irradiation at 390 mJ, and (b) wettability of stainless-steel surface after laser processing, after one month, and after six months since laser processing with different laser energies.

Video S1 shows the wettability conversion from inherent hydrophilicity to superhydrophobicity before and after the single-shot nanosecond laser processing. Moreover, enhancements of the superhydrophobicity after one month and after six months since laser irradiation are also provided.

Video S2 shows an impact test with water droplets on the aluminum surface just after laser ablation. The water was provided at the height of 3 cm to a tilted surface of 45°. The droplets were bounced off from the surface due to low surface energy.

Video S3 shows self-cleaning effects on the laser-treated and non-treated aluminum surfaces. Both surfaces were covered with white powders (cellulose) and tilted at 30°. After the water droplets touched the non-treated aluminum surface, they stayed on the surface due to the high surface energy of the aluminum substrate and mixed with the white powders. On the other hand, after the water droplets came to the laser-treated aluminum surface, they bounced off the surface. At the same time, the water droplets brought white powders together out of the laser-treated aluminum surface, making the surface itself clean.

Video S4 shows a water transportation approach using different surface energies of the laser-treated aluminum surfaces. The upper and lower aluminum surfaces were treated at different laser energies. In this experiment, a water droplet was first placed on the lower aluminum surface that was treated at a higher laser energy than the upper surface. The lower surface was moved up. Then, the water droplet touched the upper surface. When the lower surface was moved down, the upper one captured the water droplet as a "droplet tweezer" due to its higher surface energy than the lower surface. This "droplet tweezer" was then moved near another surface, which had an oil

droplet mixing with purple dye, and released the captured droplet. As a result, the captured droplet combined with the oil droplet to create a water-oil mixture.
